# Integrative analysis revealed that distinct cuprotosis patterns reshaped tumor microenvironment and responses to immunotherapy of colorectal cancer

**DOI:** 10.3389/fimmu.2023.1165101

**Published:** 2023-03-16

**Authors:** Ximo Xu, Chengsheng Ding, Hao Zhong, Wei Qin, Duohuo Shu, Mengqin Yu, Naijipu Abuduaini, Sen Zhang, Xiao Yang, Bo Feng

**Affiliations:** Department of General Surgery, Ruijin Hospital, Shanghai Jiao Tong University School of Medicine, Shanghai, China

**Keywords:** cuprotosis, colorectal cancer, tumor microenvironment, immune checkpoint inhibitor, prognosis

## Abstract

**Background:**

Cuprotosis is a novel form of programmed cell death that involves direct targeting of key enzymes in the tricarboxylic acid (TCA) cycle by excess copper and may result in mitochondrial metabolic dysfunction. However, whether cuprotosis may mediate the tumor microenvironment (TME) and immune regulation in colorectal cancer (CRC) remains unclear.

**Methods:**

Ten cuprotosis-related genes were selected and unsupervised consensus clustering was performed to identify the cuprotosis patterns and the correlated TME characteristics. Using principal component analysis, a COPsig score was established to quantify cuprotosis patterns in individual patients. The top 9 most important cuprotosis signature genes were analyzed using single-cell transcriptome data.

**Results:**

Three distinct cuprotosis patterns were identified. The TME cell infiltration characteristics of three patterns were associated with immune-excluded, immune-desert, and immune-inflamed phenotype, respectively. Based on individual cuprotosis patterns, patients were assigned into high and low COPsig score groups. Patients with a higher COPsig score were characterized by longer overall survival time, lower immune cell as well as stromal infiltration, and greater tumor mutational burden. Moreover, further analysis demonstrated that CRC patients with a higher COPsig score were more likely to respond to immune checkpoint inhibitors and 5-fluorouracil chemotherapy. Single-cell transcriptome analysis indicated that cuprotosis signature genes recruited tumor-associated macrophages to TME through the regulation of TCA and the metabolism of glutamine and fatty acid, thus influencing the prognosis of CRC patients.

**Conclusion:**

This study indicated that distinct cuprotosis patterns laid a solid foundation to the explanation of heterogeneity and complexity of individual TME, thus guiding more effective immunotherapy as well as adjuvant chemotherapy strategies.

## Introduction

Colorectal cancer (CRC) is one of the most prevalent malignancies and remains the leading cause of cancer death worldwide, with more than 30% of patients suffering from recurrence, metastasis, and death within a 5-year treatment (1, 2). Currently, immunotherapy, which makes use of immune checkpoint inhibitors (ICIs), including anti-PD-1/CTLA-4, is popular worldwide, with good results in treating non-small cell lung cancer (3, 4). Moreover, studies have demonstrated that this effective treatment has the potential to achieve a durable response in CRC as well (5, 6). Recently, the concept of tumor microenvironment (TME) has been proposed and widely appreciated as a result of the increasing knowledge of diversity and complexity of tumor components. TME is the environment where the tumor is located and various immune cells, stromal cells, extracellular matrix (ECM) molecules, and cytokines coexist (7–9). As a result of their interaction with the TME components, tumor cells show a variety of changes in biological behavior, such as the stimulation of proliferation and angiogenesis, apoptosis inhibition, and hypoxia avoidance. Emerging evidence indicates that TME appears to play a critical role in tumor progression, immune escape, and response to immunotherapy (10–12). The prediction of ICI response based on the characteristics of TME cell infiltration is a promising way to improve the current ICIs’ effect and develop new immunotherapeutic approaches (13–16). Therefore, a comprehensive analysis of different TME patterns may help identify distinct tumor immune phenotypes and further guide and predict the selection of ICIs (16, 17).

Copper is an essential metal ion that is required for many cellular functions, including energy production and antioxidant defense. However, when copper levels become excessive, it can lead to the production of reactive oxygen species (ROS) through Fenton chemistry, which can cause oxidative damage to cellular components. This oxidative stress can activate a number of cell death pathways, including apoptosis, necrosis, and autophagy, ultimately leading to cell death (18). Copper-induced cell death, also named cuprotosis, refers to the direct targeting of copper to the key lapidated enzyme of the tricarboxylic acid (TCA) cycle and thus is responsible for the dysfunction of mitochondrial metabolism (19). A great deal of progress has been made in immunometabolism in recent years, and there is substantial evidence that both the dysfunction of mitochondrial metabolism and ROS are associated with immune response (20–23). Therefore, a comprehensive understanding of whether cuprotosis is associated with TME and the response of ICIs in CRC will help deepen our understanding of it and provide new strategies for immunotherapy.

In this study, the genomic and clinical information of 1,226 CRC samples was synthesized to investigate the copper death patterns, and the correlation between the cuprotosis patterns and their related TME infiltration characteristics. Three distinct cuprotosis patterns were identified by the unsupervised consensus clustering, and we found that the TME cell infiltration characteristics of the three patterns were associated with immune-excluded, immune-desert, and immune-inflamed phenotype, respectively. Then, we established a scoring scheme to quantify the cuprotosis patterns in individual CRC patients and to predict the response to ICIs and adjuvant chemotherapy.

## Materials and methods

### Source and preprocess of publicly attainable colorectal expression datasets

The publicly attainable NCBI Gene Expression Omnibus (GEO) datasets (https://www.ncbi.nlm.nih.gov/geo/) and the Cancer Genome Atlas (TCGA) (https://cancergenome.nih.gov/) were used to retrospectively collect the gene expression and clinical characteristics of CRC samples. No further evaluation was conducted for samples who had no survival information. A total of 4 eligible CRC cohorts (GSE103479, GSE39582, TCGA-COAD, and TCGA-READ) were enrolled in this study for further analysis. As for the datasets in TCGA, RNA sequencing data of gene expression (FPKM value) were downloaded from the Genomic Data Commons (GDC) using TCGAbiolinks, an R package that was specifically developed to allow integration of GDC data (24). The FPKM values were then converted to transcripts per kilobase million (TPM) values. The “Combat” algorithm of the R package sva was used to correct the batch effect among the different datasets (25). The genomic mutation data [somatic mutation and copy number variation (CNV)] of TCGA-COAD and TCGA-READ were curated from GDC.

### Unsupervised consensus clustering for 10 cuprotosis regulation genes

Ten cuprotosis-related genes (CRGs) were extracted from the meta-cohort. Unsupervised clustering analysis was applied to identify different cuprotosis patterns and classify patients for further study based on their expression of 10 CRGs. In a consistent clustering algorithm, cluster number and the stability of each cluster are determined (26). The above steps were implemented following the ConsensuClusterPlus package, and 1,000 times repetitions were conducted to ensure the stability of classification (27).

### Functional annotation and gene set variation analysis

Gene set variation analysis (GSVA) enrichment analysis was performed by using the “GSVA” R package, in order to investigate the heterology of the different cuprotosis patterns. As a non-parametric and unsupervised approach to explore the variations in pathway and biological process activity, GSVA is generally employed in estimating the samples of an expression dataset (28). GSVA was performed using gene sets “c2.cp.kegg.v7.5.1.symbols” downloaded from MSigDB, as implemented in the GSVA package (version 1.42.0).

### Analysis of TME immune cell infiltration and the immune response predictor

ssGSEA (single sample gene set enrichment analysis) (29), EPIC (30), xCELL (31), and MCPcounter (32) algorithms were performed to quantify the relative abundance of TME immune cell infiltration as well as assess the immune function in the CRC patients. The ESTIMATE (33) algorithm was performed to estimate the immune and stromal cells in CRC. The ESTIMATE algorithm helps us predict the infiltration level of immune cells and stromal cells by calculating the immune and stromal scores. The tumor immune dysfunction and exclusion (TIDE) algorithm, including two major mechanisms of tumor immune escape, T-cell dysfunction and T-cell exclusion, was utilized to evaluate the TME and predict response to treatment with ICIs (34). Higher TIDE scores indicate a lower response rate to ICI treatment as tumor cells tend to induce immune escape.

### Identification of differentially expressed genes between distinct cuprotosis patterns

By examining the expression of 10 CRGs, we categorized samples into three different cuprotosis patterns, in order to identify CRGs. With the “limma” R package, we applied the empirical Bayesian algorithm to ascertain differentially expressed genes (DEGs) between distinct cuprotosis patterns (35). To determine the DEGs, an adjusted *p*-value < 0.05 was employed.

### Generation of COPsig sore

A COPsig score system was established to quantify the cuprotosis level of individual CRC patients. First, COP gene signatures A and B corresponded to DEGs that appeared to be positively and negatively correlated with the clusters of COP genes, respectively. Then, the dimensionality of COP gene signature A and B was reduced by performing the Boruta algorithm (36), and principal component 1 was adopted as the feature score by applying the PCA. As a last step, we determined the COPsig score group for each CRC patient using an approach similar to the gene expression grading index (37):


$$\text{COPsig}\cdot \text{score =}\sum PC1_{A}\cdot \sum PC1_{B}$$

### Cancer cell line data and chemotherapeutic response prediction

On the basis of the Genomics of Drug Sensitivity in Cancer (GDSC, https://www.cancerrxgene.org/), the largest publicly available pharmacogenomic database, we predicted the response to chemotherapy for each CRC sample. Two commonly used chemotherapy drugs, 5-fluorouracil (5-FU) and paclitaxel, were selected. Using the R package “pRRrophic”, the estimation of half-maximal inhibitory concentration (IC_50_) for each sample was determined by ridge regression, and Prediction accuracy is measured by 10-fold cross-validation based on the GDSC training dataset.

The Broad Institute-Cancer Cell Line Encyclopedia (CCLE, https://portals.broadinstitute.org/ccle/data) project compiled expression profile and mutation data of human cancer cell lines (CCLs) (38). From the Cancer Therapeutics Response Portal (CTRP) (39) and PRISM Repurposing dataset (19Q4, https://depmap.org/portal/download/Drugsensitivity), drug sensitivity data for CCLs were obtained. Based on CTRP data, 481 compounds were tested across 835 CCLs, while 1,448 compounds over 482 CCLs were contained in the PRISM dataset. In both datasets, a value for area under the curve (AUC) indicates the level of sensitivity to the treatment, with lower AUC values representing greater sensitivity. According to the suggestion of Yang et al. (40), compounds with NAs in more than 20% of samples and cell lines from hematopoietic and lymphoid tissues were excluded. Next, AUC values were imputed through K nearest neighbor (k-NN) imputation. Finally, for further analysis of CTRP and PRISM, CCLE molecular data were used.

### Genomic and clinical data collection for the ICI cohort

Four immunotherapeutic cohorts with gene expression and clinical data were enrolled in our study. Metastatic melanoma received either pembrolizumab or nivolumab (41), non-small cell lung cancer patients were administered either nivolumab or pembrolizumab (42), patients with urothelial cancer received anti-PD1/PD-L1 therapy (43), and urothelium cancers were treated with atezolizumab, an anti-PD-L1 antibody (IMvigor210 cohort) (17). The gene expression profiles were collated and transformed into TPM format for further analysis.

### Single-cell RNA sequencing analysis

GSE132257, which contained single-cell RNA sequencing (scRNA-seq) data of five CRC samples, was downloaded from the GEO database. We first filtered and standardized the scRNA-seq data using the “Seurat” R package. After standardization, the 1,500 genes with the largest variance were reserved for subsequent analysis. PCA was then conducted to reduce dimensionality of data. t-SNE was applied to sort cells into different clusters. The cell annotation of each cluster was conducted by the “SingleR” R package with reference to CellMarker (44). In order to calculate the activity of senescence-related model genes in cells, we utilized the “AUCell” R package to calculate the AUC of each cell with reference to model genes and then mapped the AUC to the corresponding cells. Cells that express more genes from the senescence-related model will exhibit higher AUC values than cells expressing fewer genes. The “NicheNet” R package was utilized to infer the interactions between epithelial cells (tumor cells) and surrounding cells (45). Genes that are expressed in larger than 10% cells of clusters were considered for ligand–receptor interactions. In paired ligand–receptor activity analysis, we extracted top 100 ligands and top 1,000 targets of DEGs of “sender cell” and “affected cell”, respectively.

### Statistical analysis

All the statistical analyses were generated by R software (version 4.1.2). All statistical *p*-values were two-sided, and *p*-value < 0.05 was considered statistically significant. A univariate Cox regression model was utilized to calculate the hazard ratios (HRs) for CRGs and cuprotosis phenotype-related genes. Patients with complete clinical information were included, and a multivariate Cox regression model was established to ascertain the independent prognostic risk factors. The results of multivariate and univariate prognostic analysis were visualized by applying the forest plot R package. Spearman correlation analysis and distance correlation analysis was used to calculate the correlation coefficient. Based on the median COPsig scores, the sample was divided into two groups: the high and low scoring groups. Differences between three or more groups were compared using one-way ANOVA and Kruskal–Wallis tests (46). The waterfall function in the maftools package was employed to display mutations in the TCGA-CRC cohort (47). Using the R package “Rcircos”, the CNV landscapes of 10 cuprotosis-regulated genes in human chromosomes were plotted.

### Cell line culture and quantitative real-time polymerase chain reaction

The CRC cell lines HT-29, HCT116, RKO, SW480, and SW620 and the normal cell line NCM460 were purchased from the China Center for Type Culture Collection (CCTCC; Shanghai, China) and cultured in RPMI-1640 (Gibco, Grand Island, NY, USA), supplemented with 10% fetal bovine serum and 1% penicillin–streptomycin (Gibco). All cells were incubated at 37°C in humidified air with 5% CO_2_. The total RNA of each cell line was extracted by FastPure^®^ Cell/Tissue Total RNA Isolation Kit V2 (Vazyme, China). Then, the NanoDrop 2000 spectrophotometer (Thermo) was used to quantify RNA. After reverse transcription of RNA to cDNA by HiScript^®^ RT SuperMix for qPCR with gDNA wiper (Vazyme, China), we performed quantitative real-time polymerase chain reaction (RT-qPCR) on cDNA using ChamQ Universal SYBR qPCR Master Mix (Vazyme, China). The cycler protocol is as follows: Stage 1: 30 s at 95°C; Stage 2: 40 cycles of 10 s at 95°C and 30 s at 60°C; and Stage 3: 15 s at 95°C, 60 s at 60°C, and 15 s at 95°C. GAPDH was exploited as an internal reference. The mRNA relative expressions of CRGs were calculated by the 2^−ΔΔCt^ method. The primer sequences used for analysis are listed in **Table S1**.

### Tissue microarray and immunohistochemistry

The CRC tissues (*n* = 80) and matched adjacent normal tissues (*n* = 80) were collected from the Department of General Surgery, Ruijin Hospital, Shanghai Jiao Tong University School of Medicine. All patients signed written informed consent before the study. The immunohistochemical assay was conducted as previously described (48). Two pathologists, blinded to clinical information, analyzed the relative intensity of specimens using ImageJ software (National Institutes of Health, USA).

### Western blotting

Proteins were electrophoresed with 4%–20% SDS-PAGE gels and transferred to polyvinylidene difluoride membranes. The membrane was blocked with 5% BSA for 1 h at room temperature and incubated overnight at 4°C in primary antibody diluent. Then, the membrane was incubated with secondary antibody for 1 h at room temperature. All bands were measured and analyzed by Quantity One software (Bio-Rad, Hercules, CA, USA). The primary antibody was anti-PDHA1 (1 µg/ml, A13687, ABclonal, CHN). The secondary antibodies such as horseradish peroxidase (HRP)-conjugated anti-rabbit (A6154) and anti-mouse (A4416) antibodies were from Sigma-Aldrich.

## Results

### Landscape of genetic variation of cuprotosis-regulated genes in colorectal cancer

**Figure 1:**
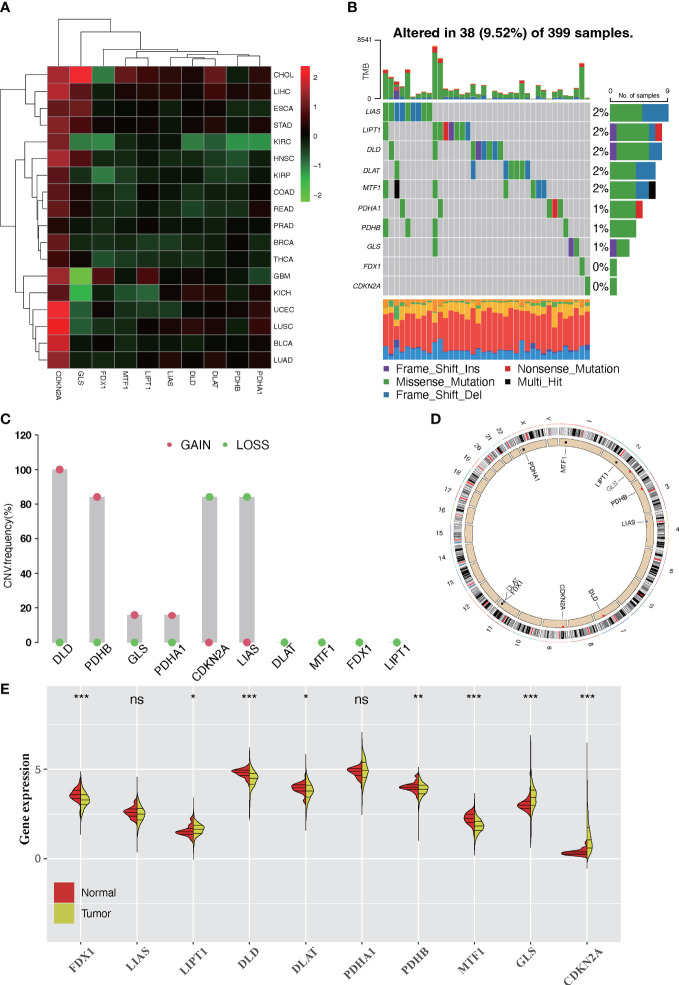
Landscape of expression and genetic alteration of cuprotosis-related genes (CRGs) in colorectal cancer. **(A)** The fold changes of the expression level alterations of CRGs in 18 cancer types, with red representing upregulated genes and green representing downregulated genes in the heatmap. **(B)** Thirty-eight of the 399 CRC patients developed genetic mutation of 10 CRGs. Each column represented an individual patient. The upper barplot showed the tumor mutational burden. The right barplot showed the frequency of each variant type. **(C)** The CNV frequency mutations of 10 CRGs. Alteration frequency was represented by the column height. Green dots represented the deletion frequency. Red dots represented the amplification frequency. **(D)** The location of CNV alteration of 10 CRGs on chromosomes. **(E)** The difference of mRNA expression level of 10 CRGs between normal and tumor CRC samples (TCGA-COAD and TCGA-READ). The asterisks represent the statistical *p*-value (ns: *p* > 0.05; *p < 0.05; **p < 0.01; ***p < 0.001).

In this study, a total of 10 CRGs were finally identified. We first examined the expression levels of 10 CRGs in pan-cancer. We found that in the majority of carcinomas, the CRGs were poorly expressed in tumors, except for CDKN2A (**Figure 1A**). Then, we demonstrated the CNV and somatic mutations of 10 CRGs in CRC. Among the 399 samples, 9.33% underwent genetic alteration of CRGs, principally including missense mutation, frame shift deletions, and nonsense mutation (**Figure S1A**). It was observed that LIAS showed the highest mutation frequency, followed by LIPT1, while neither FDX1 nor CDKN2A showed any mutation in the CRC samples (**Figure 1B**). Next, we investigated the CNV frequency mutations of CRGs, and six genes showed a CNV mutation. DLD and PDHB had a wide amplification in copy number. On the contrary, CDKN2A and LIAS were focused on the prevalent CNV deletions (**Figure 1C**). The location of CNV alterations of 10 CRGs on chromosomes is demonstrated in **Figure 1D**. Moreover, further analysis was made to investigate the mRNA expression level of CRGs between normal and CRC samples, and we found that the expressions of FDX1, DLD, DLAT, PDHB, and MTF1 were significantly decreased, whereas LIPT1, GLS, and CDKN2A were significantly upregulated in tumor samples (**Figure 1E**). The expression level of CRGs with CNV amplification was higher in CRC samples compared to normal samples (e.g., GLS and PDHA1), while the expression level of LIAS was relatively decreased in tumor samples (**Figures 1D, E**). In addition, Spearman correlation analysis was performed to evaluate the mutual regulation between the CRGs (**Figure S1B**). CDKN2A showed a significantly negative correlation with most other CRGs. The univariate and multivariate Cox model were established to ascertain whether CRGs were independent risk factors for prognosis in CRC patients. The forest plots showed that CDKN2A and GLS could be considered as a risk factor for CRC patients and correlated with a markedly shorter overall survival (**Figure S1C,D**). Thus, in the above analyses, we observed a very heterogeneous landscape of genetic and expressional changes in CRGs between normal and CRC samples. Accordingly, the imbalance in CRG expression was crucial to the occurrence and progression of CRC.

### Cuprotosis patterns mediated by 10 CRGs

**Figure 2:**
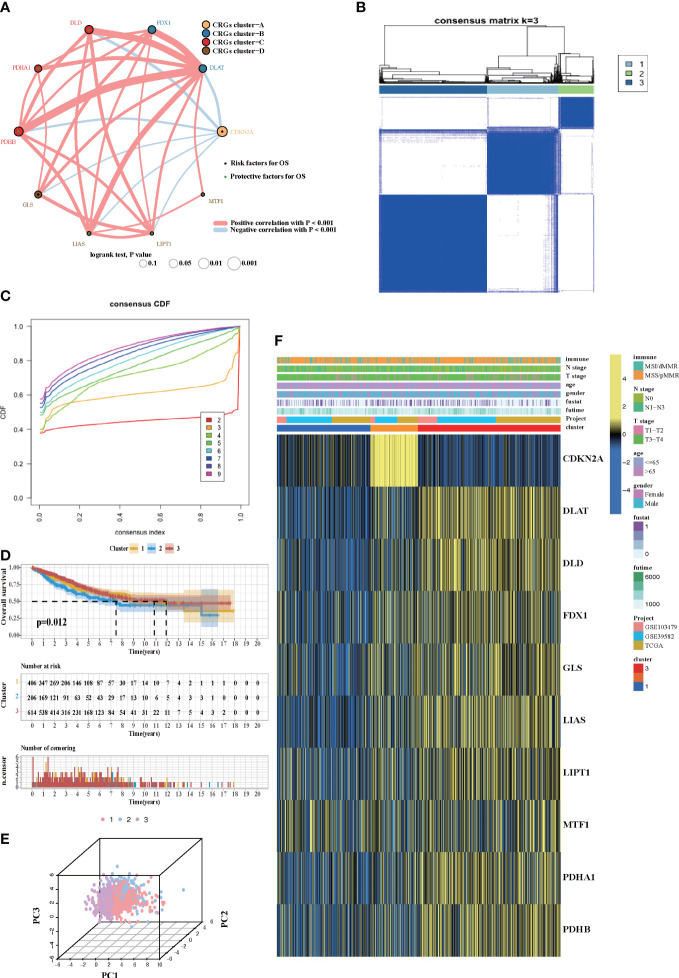
Cuprotosis patterns in CRC patients. **(A)** The network between 10 CRGs in CRC patients. The size of the circle corresponded to the effect of each gene on the patients’ prognosis, and the range of values was scaled by log-rank test. Protective factor for patients’ OS was illustrated by a green dot, and risk factors was illustrated by a black dot. The lines showed the interaction of each gene, and the thickness represented the correlation strength. Blue lines indicated negative correlation, and red lines indicated positive correlation. The CRG cluster A–D was marked with yellow, blue, red, and brown, respectively. **(B)** Unsupervised consensus clustering for 10 CRGs in the meta-cohort and the consensus matrices for *k* = 3. **(C)** Consensus values range from 0 to 1. **(D)** Kaplan–Meier curves for the three cuprotosis patterns based on 1,226 CRC patients from the meta-cohort, including 406 samples in COPcluster C1, 206 samples in COPcluster C2, and 614 samples in COPcluster C3 (log-rank test). The COPcluster C3 showed a significantly better prognosis than the other two COPclusters. **(E)** The transcriptome profiles of three cuprotosis patterns were analyzed by principal component analysis, revealing a striking difference in transcriptome profiles between different patterns. **(F)** Expression heatmap of three COPclusters of 10 CRGs in the meta-cohort. Immune subtype, age, gender, N stage, T stage, and prognosis were annotated. Yellow represents a high expression of CRGs, and blue represents a low expression.

Four datasets (GSE103479, GSE39582, TCGA-COAD, and TCGA-READ cohort) with available survival information and clinical annotations were merged in one meta-cohort. The CRG network revealed a landscape of CRG interactions, gene connection, and their prognosis significance for CRC patients (**Figure 2A**). The illustration indicated that the cross-talk among the CRGs probably plays a pivotal role in different cuprotosis patterns and was involved in CRC development and progression. Based on the above hypothesis, we stratified the samples with quantitatively distinct cuprotosis patterns according to the expression levels of 10 CRGs utilizing the R package of ConsensusClusterPlus. Three different cuprotosis patterns were eventually identified using unsupervised clustering, including 406 cases in COPcluster C1, 206 cases in COPcluster C2, and 614 cases in COPcluster C3 (**Figures 2B, C**). Then, we performed a prognosis analysis for the three main cuprotosis clusters; the results demonstrated that COPcluster C3 showed a prominent survival advantage, while COPcluster C2 was the least likely to survive in the meta-cohort (**Figure 2D**). Moreover, the unsupervised clustering discovered three totally different patterns of cuprotosis in the meta-cohort (**Figures 2E, F**). There was significant distinction in the CRG transcriptional profile among the three different cuprotosis patterns (**Figure 2F**).

### The cuprotosis patterns characterized by distinct immune landscape

**Figure 3:**
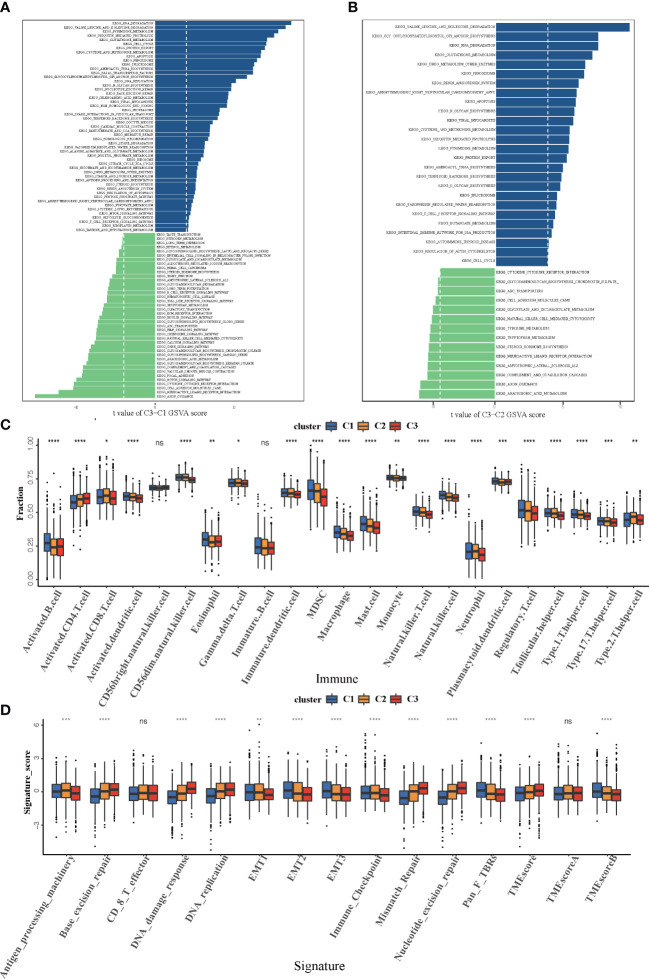
Biological and TME infiltration characteristics of each cuprotosis pattern. **(A, B)** Barplot depicting the GSVA score of representative KEGG pathways curated from MSigDB in three cuprotosis patterns. **(A)** COPcluster C3 *vs*. COPcluster C1. **(B)** COPcluster C3 *vs*. COPcluster C2. **(C)** The fraction of TME cell infiltration of three cuprotosis patterns using the ssGSEA algorithm. The top end, median line, and bottom end of the box represent the 25%, 50%, and 75% value, respectively. The black dots show outliers. The asterisks illustrate the statistical *p*-value (*p < 0.05, **p < 0.01, ***p < 0.001, ****p < 0.0001, ns *p* > 0.05). **(D)** The fraction of different signatures (immune-relevant signature, mismatch-relevant signature, and stromal-relevant signature) and TMEscore. The line in the box represents the median value. The bottom and top of the boxes are the 25th and 75th percentiles (interquartile range). The whiskers encompass 1.5 times the interquartile range. The asterisks illustrate the statistical *p*-value (*p < 0.05, **p < 0.01, ***p < 0.001, ****p < 0.0001, ns *p* > 0.05).

In order to investigate the molecular mechanisms among the three different cuprotosis patterns, GSVA enrichment analysis was performed on the KEGG gene set. We found that all the three clusters were markedly enriched in the immune signaling pathway, including the T-cell receptor signaling pathway, the B-cell receptor signaling pathway, and the Toll-like receptor signaling pathway. However, COPcluster C1 and COPcluster C2 were simultaneously enriched in stromal elements such as ECM–receptor interaction and cell adhesion molecules cams (**Figures 3A, B**). To clarify and compare the 23 immune infiltration cell subpopulations of each cluster, we then constructed a boxplot with ssGSEA. To our surprise, subpopulation analysis of TME cell infiltration indicated that the vast majority of immune cells, such as active CD4^+^ cells, eosinophils, and activated B cells, were enriched in COPcluster C1 and C3, with the least enrichment in COPcluster C2 (**Figure 3C**). Nevertheless, patients in COPcluster C3 and C2 had a longer median overall survival, while those in COPcluster C2 did not show a matching prognosis advantage (**Figure 2D**). In addition, the ESTIMATE algorithm was used to evaluate the immune cell infiltration level (Immune Score) and stromal cell infiltration level (Stromal Score) across three different cuprotosis patterns. Further analysis revealed that COPcluster C3 exhibited the lowest immune score, followed by C2 and C1 (**Figure S1E**). Meanwhile, COPcluster C1 and C2 had a much higher stromal score than COPcluster C3 (**Figure S1F**). According to previous studies, immune-excluded tumor phenotype exhibited an abundance of immune cells. Rather than penetrating tumor parenchyma, these immune cells remained in the stroma surrounding tumor cell nests (49). Thus, we hypothesized that the abundant stromal component in COPcluster C1 and C2 inhibited potential antitumor immune response. Subsequent TME analysis demonstrated that stromal activation was significantly enhanced in COPcluster C1 and C2, including the activity of epithelial–mesenchymal transition (EMT), transforming growth factor beta (TGF-β). Moreover, previous studies proposed a novel concept, TMEscore (TMEscore A − TMEscore B), representing the signature of tumor immune microenvironment (17). In our study, we found a relatively lower TMEscore B as well as a markedly higher TMEscore in COPcluster C3 (**Figure 3D**). As a result of the above findings, we confirmed that the three cuprotosis patterns developed significantly different characterizations of TME cell infiltration. COPcluster C1 was considered as an immune-excluded phenotype, characterized by diminished immune cell infiltration and stromal activation. COPcluster C2 was classified as immune-desert phenotype, characterized by immunosuppression, while COPcluster C3 was considered as an immune-infiltrated phenotype, marked by immune cell infiltration and immune activation.

We then further explored the specific correlations between CRGs and TME immune cell infiltration by Spearman’s correlation analysis (**Figure S2A**). We found that high expression of CDKN2A and MTF1 was associated with abundant immune cell infiltration, whereas PDHA1, LIPT1, LIAS, GLS, FDX1, and DLAT expression exhibited a negative correlation with the immunocyte infiltration. Among these CRGs, the relatively high level of negative correlation between PDHA1 and immune cell infiltration attracted our attention. Based on the PDHA1 expression level, the CRC samples were assigned into high- and low-expression groups according to the best cutoff of 6.68376. There was significant prognostic difference between the two groups of patients (**Figure S2B**). The results of GSVA indicated that patients with a low PDHA1 level were more likely to be associated with enrichment of immune-related signaling pathway such as natural killer cell, Toll-like receptor, and B cell response signaling pathways (**Figure S2C**). ESTIMATE algorithm was then used to quantify the overall immune cell infiltration between low and high PDHA1 samples. The results demonstrated that low expression of PDHA1 exhibited higher immune scores, which meant that the tumors with low PDHA1 expression were surrounded by more immunocyte components, thus confirming our above findings (**Figure S2D**). Additionally, we discovered that tumors with low expression levels of PDHA1 had significantly higher infiltration of 23 TME immune cells (**Figure S2E**). Furthermore, considering that PD-L1 and CTLA-4 are well-proven biomarkers for predicting the response of anti-PD-1/PD-L1 and anti-CTLA-4 treatment, we compared the expression levels of CD274 (known as PD-L1) and CTLA-4 between the different PDHA1 expression subtypes. It is not surprising that CD274 and CTLA-4 expression were significantly upregulated while the expression of PDHA1 was low (**Figures S2F, G**). Taken together, we could speculate that the PDHA1-mediated cuprotosis process might promote tumor TME immune cell infiltration, thus enhancing the intratumoral antitumor immune response. Furthermore, PDHA1 might mediate the regulation of PD-L1 and CTLA-4, thereby influencing the sensitivity of patients to immunotherapy.

### Cuprotosis phenotype-related DEGs in colorectal cancer

**Figure 4:**
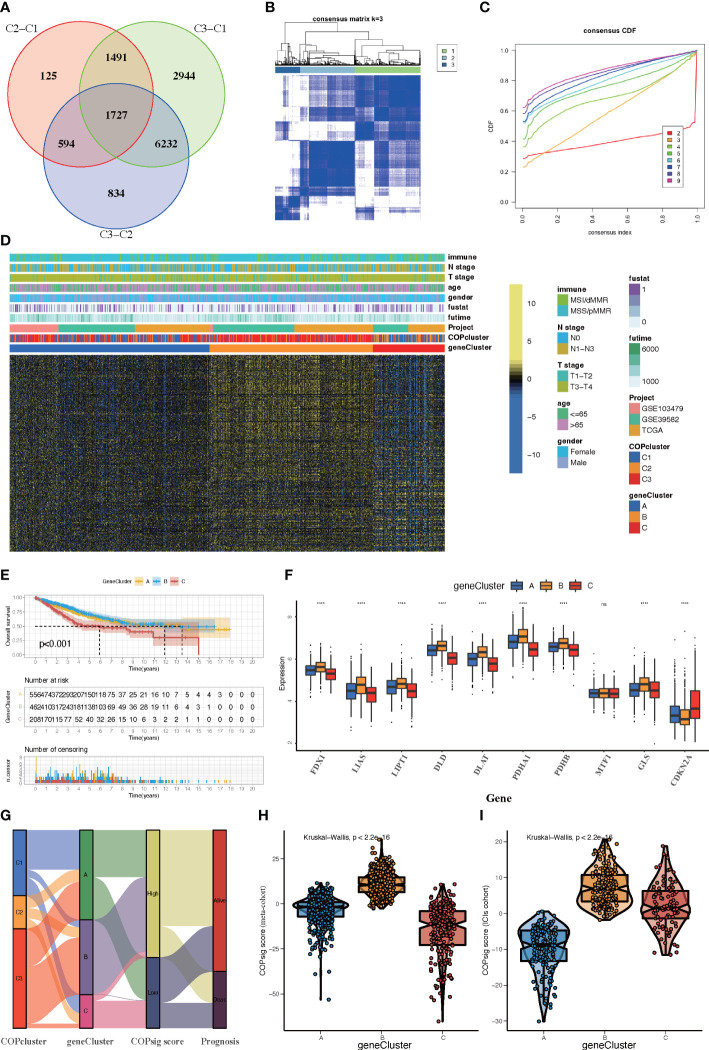
Construction of COP gene clusters and COPsig score. **(A)** A total of 1,727 cuprotosis-related differentially expressed genes (DEGs) between three cuprotosis patterns were illustrated in the Venn diagram. **(B)** Unsupervised consensus clustering for 375 prognosis-related DEGs in the meta-cohort and the consensus matrices for *k* = 3. **(C)** Consensus values range from 0 to 1. **(D)** Expression heatmap of three COP gene clusters of 375 DEGs. Immune subtype, COPcluster, age, gender, N stage, T stage, and prognosis were annotated. Yellow represents a high expression of DEGs, and blue represents a low expression. **(E)** Kaplan–Meier curves for the three COP gene clusters, including 556 samples in gene cluster A, 462 samples in gene cluster B, and 208 samples in gene cluster C (log-rank test). Gene cluster A showed a significantly better prognosis than the other two gene clusters. **(F)** The expression level of 10 CRGs in three gene clusters. The line in the box represents the median value. The bottom and top of the boxes are the 25th and 75th percentiles (interquartile range). The whiskers encompass 1.5 times the interquartile range. The asterisks illustrate the statistical *p*-value (*p < 0.05, **p < 0.01, ***p < 0.001, ****p < 0.0001, ns *p* > 0.05). **(G)** Alluvial diagram showing the changes in COPclusters, gene clusters, COPsig score, and patients’ prognosis. **(H, I)** Differences in COPsig score among three COP gene clusters in the meta-cohort **(H)** and the ICI cohort **(I)**. The Kruskal–Wallis test was used to compare the statistical difference between three gene clusters (*p* < 0.001).

Although samples were classified into three different cuprotosis patterns, the potential genetic alterations and expression disturbances in these phenotypes remained unclear. Based on these queries, we further investigated the underlying cuprotosis-related transcriptional expression change across three cuprotosis patterns in CRC. For each of the three cuprotosis patterns, the Limma package was applied to identify overlapping DEGs. A total of 1,727 DEGs representing the crucial distinct indices of the three cuprotosis patterns were selected and illustrated in the Venn diagram (**Figure 4A**). Afterwards, in order to screen for DEGs associated with patients’ prognosis, a univariate Cox analysis was performed, which resulted in 375 genes. To further validate the biological process of CRGs, we subsequently performed an unsupervised clustering analysis based on the selected 375 CRGs to classify the samples into different genomic subtypes. The stratifications assigned samples into three subgroups consistent with the clustering grouping of cuprotosis patterns, and we named the three distinct subgroups COP gene clusters A–C (**Figures 4B-D**). The results demonstrated that three different cuprotosis patterns did exist in CRC. We found that patients with relatively advanced T stage and N stage were probably represented by COP gene cluster C, while patients with MSI/dMMR were more likely to be characterized by COP gene clusters A and B (**Figure 4D**). Among 1,226 colorectal patients, 556 were found to be clustered in the COP gene cluster A, which was linked to a better prognosis. While a worse survival outcome was observed for patients in gene cluster C, an intermediate prognosis was observed in gene cluster, with a total of 462 patients aggregated (**Figure 4E**). The expression level of the 10 CRGs among the three gene clusters was compared and is shown in **Figure 4F**. We observed significant differences in CRG expression between the three gene clusters, which was also in accordance with the expected results of cuprotosis patterns.

### Construction of COPsig score and exploration of its clinical relevance

**Figure 5:**
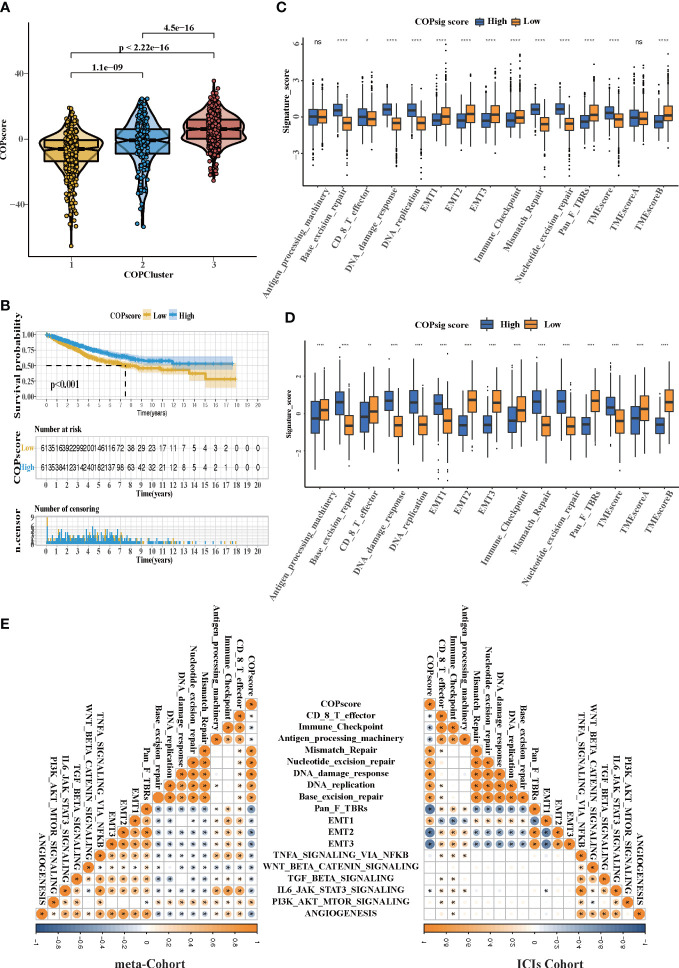
The TME cell infiltration characteristics in the high and low COPsig score groups. **(A)** Differences in COPsig score among three COPclusters in the meta-cohort. The Kruskal–Wallis test was used to compare the statistical difference between three gene clusters (*p* < 0.001). **(B)** Kaplan–Meier curves for the two COPsig score groups, including 789 samples in the high COPsig score group, and 437 samples in the low COPsig score group (log-rank test). The high COPsig score group showed a significant better prognosis. **(C)** The fraction of TME cell infiltration of the high and low COPsig score groups using the ssGSEA algorithm. The top end, median line, and bottom end of the box represent the 25%, 50%, and 75% values, respectively. The black dots show outliers. The asterisks illustrate the statistical *p*-value (*p < 0.05, **p < 0.01, ***p < 0.001, ****p < 0.0001, ns *p* > 0.05). **(D)** The fraction of different signatures (immune-relevant signature, mismatch-relevant signature, and stromal-relevant signature) and TMEscore. The line in the box represents the median value. The bottom and top of the boxes are the 25th and 75th percentiles (interquartile range). The whiskers encompass 1.5 times the interquartile range. The asterisks illustrate the statistical *p*-value (*p < 0.05, **p < 0.01, ***p < 0.001, ****p < 0.0001, ns *p* > 0.05). **(E)** Correlations between COPsig score and the known biological gene signatures in the meta-cohort and the ICI cohort using Spearman analysis. Negative correlation was marked with blue and positive correlation was marked with orange.

Despite our findings indicating that cuprotosis patterns were involved in prognosis and immune infiltration, these analyses are based only on patient populations and cannot accurately predict the signatures of cuprotosis in individual tumors. We thus formulated a scoring scheme known as the COPsig score, which hinged on the identified cuprotosis-related signature genes, to classify the patterns of cuprotosis in individual colorectal patient. Due to the complexity of cuprotosis quantification, an alluvial diagram could be used to illustrate the workflow of COPsig score construction (**Figure 4G**). Meanwhile, we calculated the COPsig score in the ICI cohort in the same manner, to confirm our results. Kruskal–Wallis test revealed a prominent difference between COPsig score and COP gene clusters. Gene cluster B showed a higher median COPsig score, indicating that high COPsig scores were likely to be associated with immune activation-related signatures, whereas gene clusters A and C illustrated a relatively lower median COPsig score (**Figures 4H, I**). In particular, COPcluster C3 had a significantly higher COPsig score compared to other clusters and COPcluster C1 presented the lowest COPsig score (**Figure 5A**). We then ought to ascertain the prognostic capacity of the COPsig score to predict oncological outcomes by assigning patients into high or low scoring groups with a cutoff of 0.658 (see *Materials and Methods*). As anticipated, patients with a high COPsig score were markedly related to a better prognosis (**Figure 5B**). As an additional step in validating the COPsig score, we used the three CRC cohorts mentioned above to determine the relationship between the COPsig score and patient prognosis (GSE103479, GSE39582, and TCGA-CRC). In a similar manner to the results above, high COPsig scores were significantly correlated with better survival outcomes (**Figures S3A–C**). Based on the univariate and multivariate Cox regression model analysis considering patient age, gender, T stage, N stage, and COPsig score, COPsig score was found to serve as a reliable and independent protective factor for assessing patient survival outcomes (**Figures S3D, S4E**). According to the analysis of the relevant components of TME, a significant association was revealed between low COPsig score and stromal-related pathways in both the meta-cohort and the ICI cohort (**Figures 5C, D**). To better characterize the cuprotosis signature, we also examined the correlation between the signatures and COPsig scores. According to the heatmap of correlation matrix, COPsig score was negatively correlated with immune activation process, EMT, and stromal-related features, but was positively correlated with DNA repair signatures in both the meta-cohort and the ICI cohort (**Figure 5E**). Moreover, the ESTIMATE algorithm was used to further examine the immune characteristics of high and low COPsig scores. We could find that in both the meta-cohort and the ICI cohort, low COPsig scores were strongly associated with high immune scores and stromal scores (**Figures S4A–D**). Next, we performed xCell, MCPcounter, single-sample gene set enrichment analysis (ssGSEA), and EPIC algorithm, in order to illustrate the immune landscape of high and low COPsig scores. As shown in **Figure S4E, F**, abundant immune cell infiltration could be found in the low COPsig score group, and the level of immune infiltration was negatively correlated with the COPsig scores. In light of the above findings, low COPsig scores were significantly associated with immune activation and stromal activation. The COPsig score could be used to distinguish individual colorectal tumors’ patterns of cuprotosis and further characterize the TME immune cell infiltration. In addition, high COPsig scores were strongly correlated with better survival outcomes, creating an accurate predictor of CRC patient prognosis.

Then, using the Maftools package, the distribution differences of somatic mutation between high and low COPsig scores in the TCGA-CRC cohort were analyzed. The high COPsig score group had a greater tumor mutation burden than the low COPsig score group. Mutational landscapes revealed that APC (81% *vs*. 62%) and tp53 (58% *vs*. 45%) were more susceptible to somatic mutations in the high COPsig score group (chi-square test, *p* < 0.05, **Figures S3F, G**). The TMB quantification analysis supported the hypothesis that high COPsig score tumors correlated markedly with a higher TMB (**Figure S3H**). Increasing evidence indicated that patients with high TMB status appear to respond to immunotherapy with durable clinical effects. In summary, the above results inferred that the differences in tumor cuprotosis patterns might act as a critical factor mediating clinical responses to immunotherapy. The COPsig scores were found to be able to indirectly predict immunotherapy, as well.

### Implications of COPsig scores in the prediction of immune response and drug sensitivity

**Figure 6:**
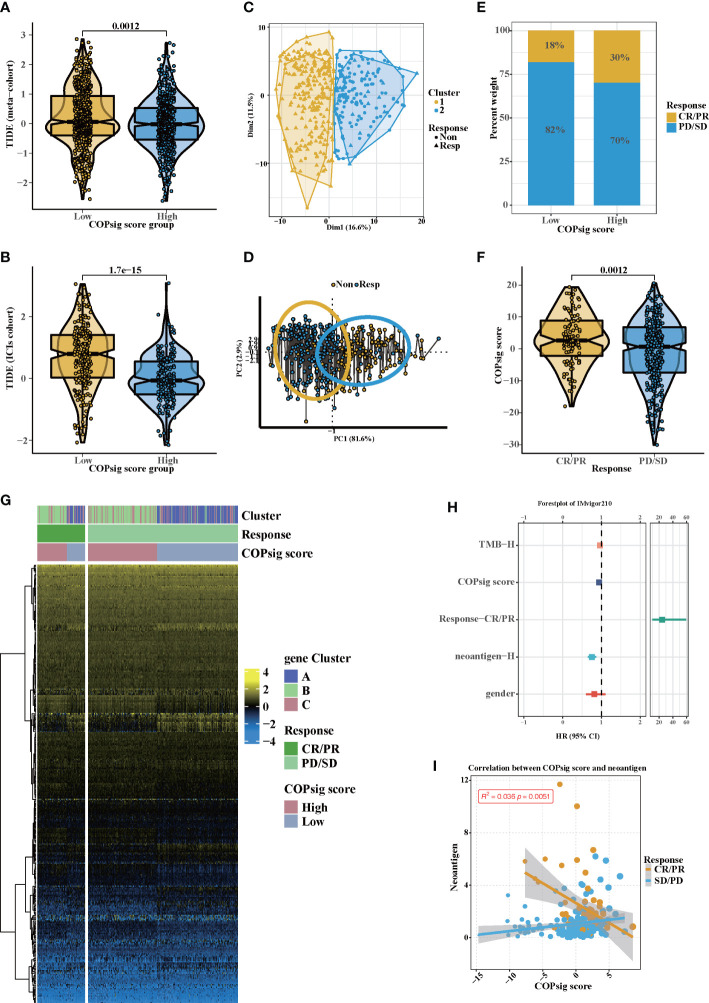
Potential immunotherapy in high and low COPsig score group. (A, B) The relative distribution of TIDE was compared between COPsig score high vs. low groups in meta-cohort **(A)** and ICIs cohort **(B)**, respectively. **(C, D)** Principal component analysis of the ICIs cohort and the response to ICIs. **(E, F)** The fraction of patients with immunotherapy response (ICIs cohort) in low and high COPsig score groups **(E)**. The COPsig score of CR/PR and PD/SD patients in ICIs cohort **(F)**. **(G)** Expression heatmap in meta-cohort. Gene clusters, response, COPsig score, were annotated. Yellow represented a high expression, and blue represented a low expression. **(H)** Univariate cox regression model estimating clinical prognosis significance between TMB, COPsig score, response, neoantigen and gender. **(I)** correlation between COPsig score and neoantigen in CR/PR. Yellow represented CR/PR patients, blue represented SD/PD patients.

There is no doubt that anti-CTLA-4/PD-1 therapy has made a significant breakthrough in antitumor therapy. TIDE, a newly identified immune response predictor, is widely used and is strongly recommended in addition to some of the well-known TMB, PD-L1, and MSI measures (50, 51) According to our analysis, both in the meta-cohort and the ICI cohort, the TIDE value significantly declined in the high COPsig group (*p* < 0.01 in the meta-cohort, *p* < 0.001 in the ICI cohort, **Figures 6A, B**). It appeared from these findings that the expression of tumor-specific cuprotosis patterns played a critical role in regulating immune responses. As COPsig score offered a robust correlation with immune response, we next investigated whether COPsig score could predict patient response to ICI therapy in four immunotherapy cohorts. We found that in the ICI cohort, patients were assigned into two groups (**Figures 6C, D**). Patients with a high COPsig score were proven to have significant benefit and immune response to ICI treatment (response rate: 30% *vs*. 18%, **Figure 6E**). **Figure 6F** also illustrates that patients who received CR/PR tended to have a higher COPsig score (*p* = 0.0012). A heatmap illustrated the gene expression difference between the high and low COPsig score groups, which might be correlated with the response of ICIs (**Figure 6G**). Then, we established a univariate Cox model to predict whether COPsig score was an impact factor for patient prognosis (**Figure 6H**). The results showed that COPsig score, neoantigen, TMB, and gender were protective factors, while response to ICIs was a risk factor for patients’ long-term survival. Moreover, for patients who benefited from ICIs, COPsig score was significantly negatively correlated with neoantigen expression, while COPsig score was positively correlated with neoantigen expression in the PD/SD group, which confirmed the above results (**Figure 6I**).

**Figure 7:**
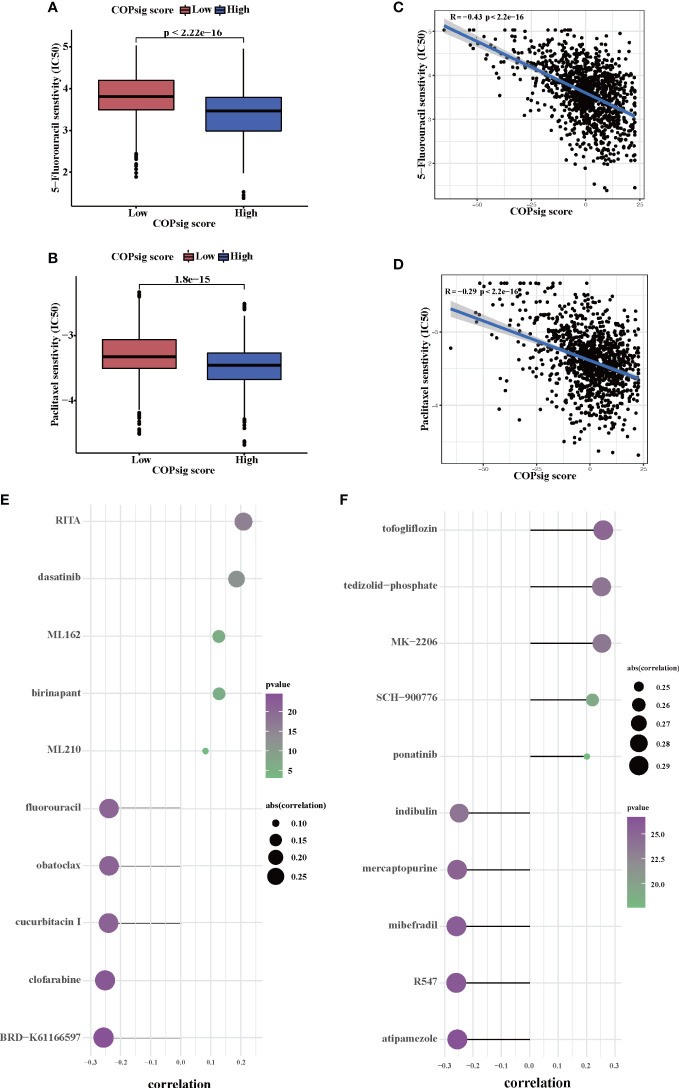
Chemotherapy response in the high and low COPsig score groups. **(A, B)** The differences of response to 5-FU **(E)** and paclitaxel **(F)** between the high and low COPsig score groups. **(C, D)** The correlation between COPsig scores of patients and the estimated IC_50_ value of 5-FU **(C)** and paclitaxel **(D)**. **(E)** The results of Spearman’s correlation analysis of 10 CTRP-derived compounds. **(F)** The results of Spearman’s correlation analysis of 10 PRISM-derived compounds.

Since chemotherapy is the most common form of treatment for CRC, we assessed the effect of two chemo drugs: 5-FU and paclitaxel. The ridge regression model was then trained by ridge regression on the GDSC cell line dataset and proven accurate by 10-fold cross-validation. Based on our predictive models of these two drugs, we estimated the IC_50_ for each sample in the meta-cohort. There was a significant difference between low and high COPsig scores for the two chemo drugs, with the high COPsig score group being more sensitive to commonly administered chemotherapy (*p* < 0.001 for 5-FU and paclitaxel, **Figures 7A, B**). Furthermore, the correlation analysis demonstrated that the IC_50_ values of both drugs were markedly negatively correlated with the COPsig score as well (**Figures 7C, D**).

Then, a drug response prediction model was built using the CTRP and PRISM datasets that contain gene expression profiles and drug sensitivity profiles for hundreds of CCLs. Compounds with NAs in more than 20% of samples and cell lines from hematopoietic and lymphoid tissues were excluded. Moreover, NAs were filled using the k-NN algorithm. Ultimately, the analysis was then carried out using 680 CCLs (containing 354 compounds) from the CTRP dataset and 480 CCLs (containing 1,285 compounds) from the PRISM dataset, respectively. Afterwards, in order to predict the response for each compound in each sample, the pRRophetic package with the ridge-regression model was utilized to obtain an estimated AUC value based on the expression profile. Next, the correlation between AUC values and COPsig scores was analyzed using Spearman correlation analysis, and we select the compounds with the top five and the bottom five Spearman’s *r* value in CTRP and PRISM datasets, respectively (**Figures 7E, F**). We found that 5-FU showed a higher drug sensitivity in high COPsig score patients, which further confirmed the above results. In general, our results strongly indicated that the COPsig score had a direct link with the response to immunotherapy and chemotherapy.

### Cuprotosis signature genes in single-cell transcriptomic data

**Figure 8:**
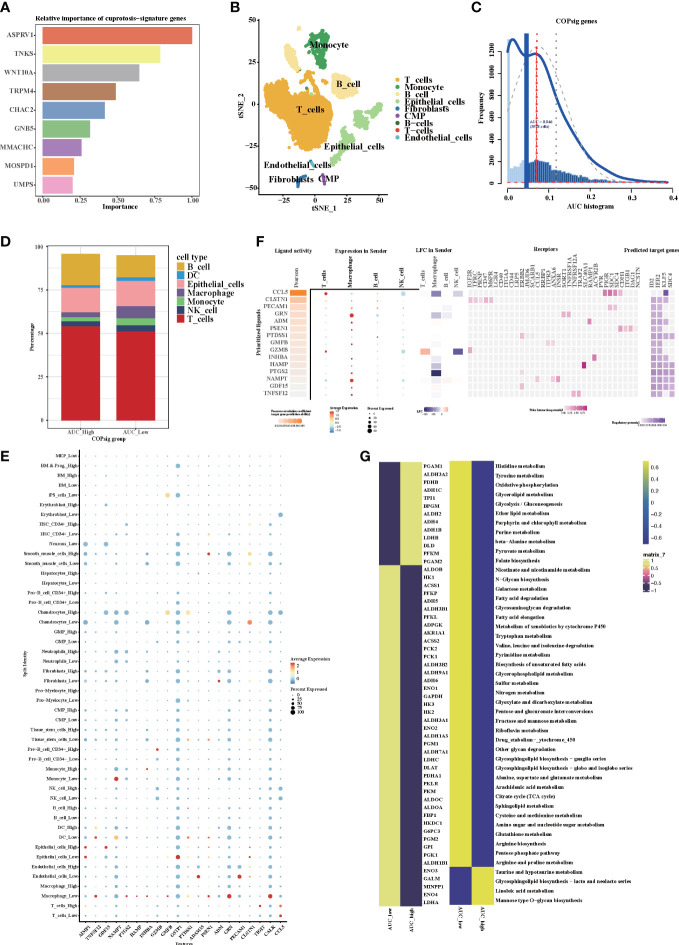
The expression of cuprotosis signature genes in TME by single-cell transcriptome analysis. **(A)** Relative importance of cuprotosis signature genes analyzed by random forest algorithm. **(B)** The t-SNE (t-distributed stochastic neighbor embedding) plot of 6,490 cells grouped into 12 clusters. **(C)** The threshold was chosen as 0.046 and the AUCell score of 3,918 cells exceeded the threshold value. **(D)** Percentage of each distinct cells in the high and low AUCell score groups. **(E)** Heatmap shows the expression of the top 20 active ligands in cells of the high and low AUCell score groups. The size of the dot represents the percent expressed. Red represents high expression; blue represents low expression. **(F)** Expression of the first 15 active ligands in different cells, as well as their interacting receptors and downstream potential target genes. **(G)** Metabolic differences in the high and low AUCell score groups.

Random forest algorithm was utilized to screen out the top nine important genes among the cuprotosis signature genes for further analysis (**Figure 8A**). After rigorous data normalization and filtering, 6,490 cells were retained for further analysis. In the following step, we used graph-based clustering to separate the cells into 12 clusters after normalizing them using principal component analysis (**Figure S5A**). These clusters can be assigned to cell lines by marker genes or DEGs (**Figures 8B**, **S5B**). According to the AUC values, two peaks of all cells were observed, whereas 3,918 cells had relatively higher AUC values (**Figures 8C**, **S4C**). The stacking map showed that there were more macrophages in the AUC_low group, which were consistent with our results of bulk RNA-seq analysis (**Figure 8D**). Moreover, GSVA indicated that cell adhesion pathways and immune-related pathways were enriched in the AUC_high group, which further confirmed the results of bulk RNA-seq analysis (**Figure S5D**). We next used CellChat and NicheNet to identify the expression of ligands at different cell interfaces and thus predict the cross-talk of the top 15 active ligand and relative receptors (**Figures 8E, F**). The results indicated that TNFSF12 interacted with TNFRSF12A on macrophage cells and thus potentially targeted ID2, IER2, and SDC4. In addition, interactions related to cell adhesion such as MDK–SDC4 and cytokine interactions such as CXCL2–CXCR4 were observed (**Figure S5E**). Previous studies have confirmed that cytokines such as CXCL2 and CXCR4 can recruit macrophages (52–54). Therefore, we hypothesized that cuprotosis signature genes might affect TME through the recruitment of macrophages, thereby influencing the prognosis of colorectal patients and the response to immunotherapy. Since cuprotosis influences the TCA cycle, we then explored the difference in metabolism pathways between AUC_high and AUC_low groups. As illustrated, TCA-associated genes observed a preference correlated with the AUC_low group, and enriched TCA pathway, glycolysis pathway, and oxidative phosphorylation pathway could be found in the AUC_low group (**Figures 8G**, **S5F, G**). Taken together, our findings indicate that cuprotosis signature might recruit macrophages and thus developed interaction networks with surrounding cells, which potentially induced cellular senescence and promoted the remodeling of the TME.

### PDHA1 was downregulated in CRC and associated with worse prognosis

**Figure 9:**
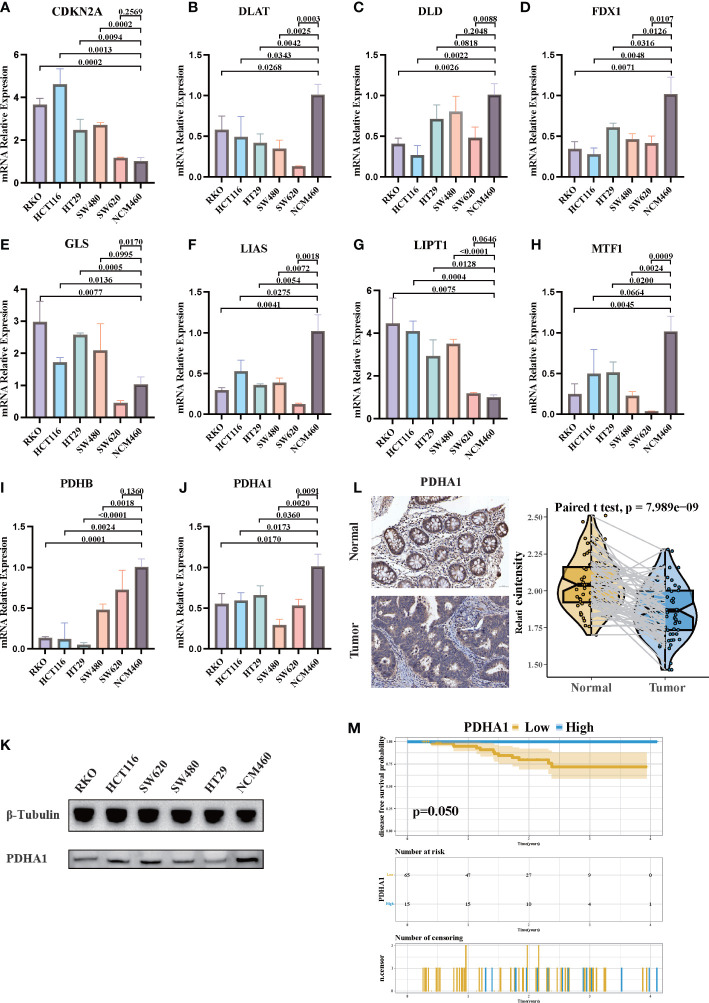
The expression of CRGs in CRC cell lines and tissues. **(A-J)** The mRNA relative expression of each CRG in 5 CRC cell lines and normal colon cell line. The asterisks illustrated the statistical *p*-value. **(K)** Western blotting results of PDHA1 protein levels in five CRC cell lines and the normal colon cell line. **(L)** PDHA1 was downregulated in colorectal cancer tissues compared to normal tissues, as examined by immunohistochemistry. **(M)** Kaplan–Meier analysis of the disease-free survival rate of CRC patients, which is stratified according to the expression of PDHA1.

Since cuprotosis patterns might influence the prognosis of CRC patients, RT-PCR was performed to examine the relative expression of CRGs in CRC cell lines and the normal cell line (**Figures 9A-J**). Similar to our previous results, the mRNA expressions of CDKN2A, GLS, and LIPT1 were upregulated in CRC, whereas the expressions of other CRGs were downregulated. As a crucial gene in the glucose metabolism reprogram of tumor cells, there is growing evidence that PDHA1 might act as a prognostic and immune-related biomarker and negatively associated with immune cell infiltration in TME (55). Western blotting confirmed that PDHA1 expression in normal colon cells was higher than that in CRC cells (**Figure 9K**). In order to confirm the relationship between PDHA1 and prognosis of CRC patients, we enrolled 80 CRC patients from our center. The expression level of PDHA1 was examined by immunohistochemistry. Compared with normal tissues, the expression level of PDHA1 in tumor tissues was significantly lower (**Figure 9L**, *p* < 0.001). The tumor samples were then divided into PDHA1-high (*n* = 15) and PDHA1-low (*n* = 65) groups according to the relative intensity. Kaplan–Meier analysis indicated that CRC patients in the PDHA1-low group had a lower disease-free survival rate (**Figure 9M**). Our findings further validated the results of Bulk-RNAseq analysis and demonstrated that PDHA1 was a potential prognostic biomarker for CRC patients.

## Discussion

Copper-induced cell death, also named cuprotosis, is a novel discovered type of programmed cell death, which refers to the direct binding of copper to the lipoylated proteins of the TCA cycle, further inducing mitochondrial dysfunction and ROS accumulation (18, 19). Mounting evidence has shown that not only mitochondrial dysfunction and ROS accumulation, but also programmed cell death pathway, are associated with the TME and immune response (20–22, 56). Therefore, clarifying the role of cuprotosis patterns in TME cell infiltration could shed light on the mechanism of cuprotosis patterns in antitumor immune responses, as well as facilitate an effective immunotherapy strategy.

In the present study, we examined the 10 CRGs and identified three different cuprotosis patterns. Distinct patterns of TME cell infiltration characteristics can be distinguished through these three patterns. COPcluster C1 was considered as an immune-excluded phenotype, characterized by the presence of abundant immune cell and stromal infiltration, together with EMT and TGF-β signaling pathway activation. COPcluster C2 was classified as the immune-desert phenotype, characterized by immunosuppression. COPcluster C3 was considered as an immune-infiltrated phenotype, marked by immune cell infiltration and immune activation. Lots of evidence have reported that TME, particularly the infiltrating immune and stromal cells, are strongly correlated to tumor progression and immunotherapeutic response (14, 57, 58). The presence of immune cells such as CD4+/CD8+ T-cell infiltrating tumors is correlated with the likelihood of an immune response (59). Conversely, immune cells can be surrounded by a dense stroma, maintaining a nest around tumor cells instead of penetrating the parenchyma. This weakens the antitumor immune response. The antitumor immune response is thus diminished. Moreover, recent studies have provided evidence that the infiltration of lymphocytes into tumor parenchyma is hindered by activation of EMT and TGF-β pathways (60, 61). Collectively, our findings were consistent with the above definitions, which corroborated the accuracy of our immunophenotype classification of the three cuprotosis patterns. Meanwhile, we speculated that CRC patients with COPcluster C3 patterns might benefit from ICI treatment and have a better prognosis.

Moreover, in the present study, differences in mRNA transcriptome between distinct cuprotosis patterns have been demonstrated to be significantly associated with immune-related biological pathways. The DEGs were considered as cuprotosis-related signature genes. In accordance with the results of cuprotosis pattern clustering, three genomic clusters based on cuprotosis signature genes were identified and strongly correlated with different prognosis and TME landscapes. A comprehensive evaluation of cuprotosis modification patterns will help us to better understand the infiltration features of TME cells and thus predict the response to immunotherapy. Therefore, in order to provide more accurate guidance on individual treatment strategies, we developed a quantitative system called “COPsig score” to identify different cuprotosis patterns. The results indicated that the cuprotosis patterns characterized by the immune-excluded phenotype showed a lower COPsig score, while the pattern characterized by the immune-inflamed phenotype had a higher COPsig score. Further analysis elucidated that COPsig score was an independent prognosis biomarker in CRC. According to recent studies, patients with low TIDE scores and high TMB are more likely to benefit from ICIs, while EMT and TGF-β pathway activation might play a critical role in resistance to ICIs (34, 60, 62, 63). The activation of EMT and TGF-β pathways, higher stromal scores, higher TIDE scores, and lower TMB were found in the low COPsig score group. Indeed, in the four independent ICI cohorts, the COPsig score was confirmed to be valuable for predicting the response to immunotherapy. The COPsig score showed a significant difference between responders and non-responders.

5-FU is an anti-metabolic drug with substitution of fluorine for hydrogen at the C-5 position of uracil, which has been broadly used since 1957 for the treatment of different types of cancer (64). To improve the efficacy and reduce toxic effects, 5-FU is often used in combination with other chemotherapeutic agents. Some studies have shown that combination chemotherapy with 5-FU can significantly prolong the survival time and relieve symptoms of CRC patients. For example, a randomized controlled trial of 423 CRC patients showed that combination treatment with 5-FU and oxaliplatin can significantly prolong the progression-free survival and overall survival of patients (65). Another clinical study of 572 patients with advanced CRC also showed that combination treatment with 5-FU and irinotecan can significantly improve the survival rate and relieve symptoms (66). However, the response rate to 5-FU-based chemotherapy is still low and the development of chemoresistance often hampers the benefit of the therapy (67, 68). Hence, the identification and validation predictive biomarkers for 5-FU-based chemotherapy might improve the prognosis of CRC patients in the future. Interestingly, recent studies have found that the activation of ferroptosis is associated with chemosensitization to 5-FU (69). We speculated that copper-induced cell death, as a type of programmed cell death as well, might be associated with the chemosensitivity of 5-FU. Our results indicated that 5-FU showed a higher drug sensitivity in high COPsig score patients in two datasets. The findings above substantiated our speculation that cuprotosis patterns could potentially be employed in clinical practice to pinpoint immune phenotypes and guide therapeutic strategies.

Tumor-associated macrophages (TAMs) in TME promote tumor development, invasion, metastasis, immune suppression, angiogenesis, and drug resistance, thereby affecting patient prognosis and playing a crucial role in regulating complex immune responses (70–73). In our study, the expression level of cuprotosis signature genes was related to the number of TAM infiltrations. Moreover, the expression of TNFSF12 was higher in the low AUCell score group, associated with tumor proliferation, invasion, migration, and angiogenesis (74). Potentially targeted gene SDC4, a transmembrane heparan sulfate proteoglycan, is considered as a central mediator of growth factors, ECM molecules, and cytoskeletal signaling proteins (75–77). Furthermore, it seems that SDC4 might be a valuable target for cancer diagnosis and treatment, since it is significantly reduced by trastuzumab and panitumumab (78, 79). Research has shown that metabolism can regulate the differentiation, mobilization, and function of TAMs, such as the glycolysis process leading to the recruitment of macrophages and polarization towards the M2 phenotype (80). Furthermore, M2 TAMs are associated with fatty acid and glutamine metabolism (81). According to our results, it was proposed that cuprotosis signature genes influenced TCA and increased glutamine and fatty acid metabolism, thereby recruiting M2 TAMs to TME and influencing the prognosis of CRC patients.

The PDHA1 gene encodes the alpha subunit of the human pyruvate dehydrogenase complex, which plays a crucial role in catalyzing the conversion of pyruvate to acetyl-CoA, an important step in the citric acid cycle and a major pathway for cellular energy production. There is a complex relationship between the PDHA1 gene and cancer. On the one hand, the PDHA1-encoded pyruvate dehydrogenase complex plays an important role in energy metabolism, and the expression level of PDHA1 is closely associated with cell proliferation and energy metabolism in some cancers, suggesting that it may promote tumor growth and metastasis. On the other hand, mutations or deletions of the PDHA1 gene have also been found, suggesting that PDHA1 may act as a potential tumor suppressor gene. Several studies have found that the expression level of the PDHA1 gene is elevated in various cancers such as ovarian cancer, and high expression levels are closely related to the malignancy and prognosis of tumors. In addition, the PDHA1 gene is involved in regulating the oxidative stress response of tumor cells, enabling them to acquire stronger antioxidant capabilities and survival advantages, thereby promoting tumor cell growth and metastasis (82, 83). Some studies have also suggested that the PDHA1 gene may act as a potential tumor suppressor gene in gastric and renal cell carcinoma, and its mutations or deletions can lead to disruptions in cellular energy metabolism and inhibition of autophagy and apoptosis, and promote tumor formation and development (84, 85). However, the relationship between PDHA1 and the prognosis of CRC patients is still unclear. In our study, we found that PDHA1 might act as a tumor suppressor in CRC. The lower expression level of PDHA1 was consistent with worse prognosis of CRC patients. Furthermore, lower PDHA1 expression is associated with higher PD-L1 and CTLA-4 expression levels, as well as increased immune cell infiltration, suggesting that PDHA1 may be involved in the remodeling of the colorectal TME, and may therefore affect the efficacy of immune therapy.

Taken together, the COPsig score could be clinically applied for the comprehensive evaluation of the cuprotosis patterns and the corresponding TME infiltration characteristics in individual CRC patients. Thus, it is possible to determine the immunophenotype of the tumor and devise a more effective therapeutic strategy. Moreover, as an independent prognostic biomarker, the COPsig score could predict not only patient survival but also the response to adjuvant chemotherapy and immunotherapy. Furthermore, we found that by changing the cuprotosis patterns, the TME infiltration characteristics subsequently changed, which was the transformation of immune-excluded and immune-desert phenotypes to the immune-inflamed phenotype, thus improving the response to ICIs.

The limitations of this study should not be neglected. First, although we reviewed the literature and selected 10 genes recognized as CRGs, other potential genes may exist since the concept of cuprotosis was somewhat novel and there are few relevant studies. Second, the evidence level of our study was relatively low due to the retrospective nature of the ICI dataset as well as the absence of appropriate ICI-based CRC datasets. Third, the follow-up time of enrolled CRC patients was relatively short, as 18 out of 65 PDHA1-low patients had less than 1 year of follow-up, which resulted in imperfect results of Kaplan–Meier analysis.

## Conclusion

Collectively, our works led to a better understanding of the regulation mechanisms underlying cuprotosis patterns on CRC TME cell infiltration. The distinct cuprotosis patterns laid a solid foundation to the explanation of heterogeneity and complexity of individual TME, thus guiding more effective immunotherapy as well as adjuvant chemotherapy strategies.

## Data availability statement

The datasets presented in this study can be found in online repositories. The names of the repository/repositories and accession number(s) can be found within the article/**Supplementary Materials**.

## Ethics statement

The studies involving human participants were reviewed and approved by Committee of Ruijin Hospital. The patients/participants provided their written informed consent to participate in this study.

## Author contributions

XX, CD, HZ and WQ performed the bioinformatic analysis. DS, MY and NA checked the manuscript and the language. SZ, XY and BF designed the study. All authors contributed to the article and approved the submitted version.
