# Supplementary material for: Integrative analysis revealed that distinct cuprotosis patterns reshaped tumor microenvironment and responses to immunotherapy of colorectal cancer
Source: Front Immunol. 2023 Mar 16;14:1165101. doi: 10.3389/fimmu.2023.1165101 (PMC10060625; doi:10.3389/fimmu.2023.1165101)
Supplement: Supplementary file 1 [file DataSheet_1.docx]

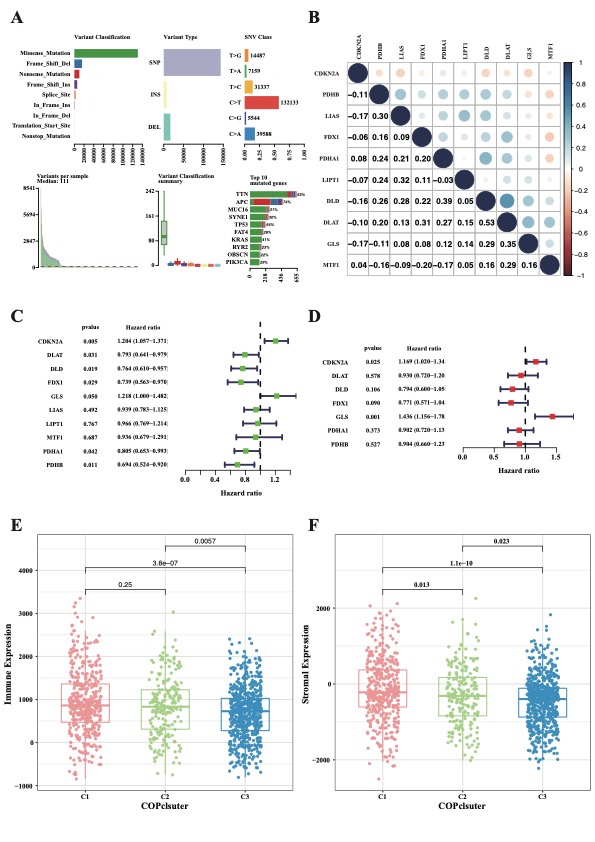


**Figure S1.** Overview of the characteristics of cuprotosis patterns. **A.** Overview of TGCA CRC cohort mutations. **B.** correlation heatmap between the 10 CRGs in CRC patients using Spearman correlation analysis. Red represented positive correlation; blue represented negative correlation. **C-D.** Univariate (**C**) and multivariate (**D**) cox regression model estimating clinical prognosis significance between 10 CRGs. **E-F.** ﻿The immune score (**E**) and stromal score (**F**) of three COPclusters in meta-cohort.


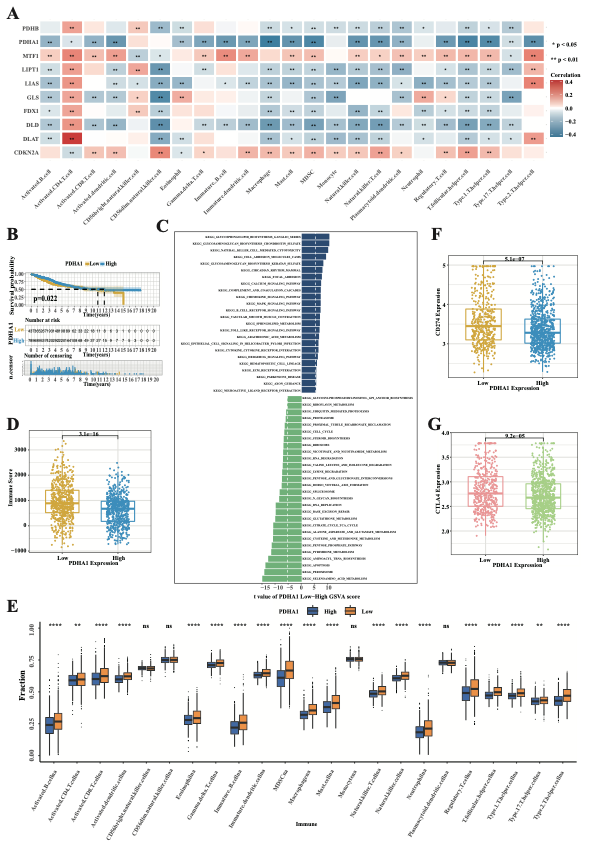


**Figure S2.** Correlation between TME infiltration and CRGs and the roles of PDHA in CRC. **A.** The correlation between each TME infiltration cell type and each CRG using spearman analysis. **B.** Survival analyses for patients with low or high PDHA1 expression in meta-cohort. **C.** Barplot depicting the GSVA score of representative KEGG pathways curated from MSigDB in PDHA1 high and low groups. **D.** The immune score (**D**) of PDHA1 high and low groups. **E-F**. Difference of CD274 (**E**) and CTLA4 (**F**) expression level between PDHA1 high and low groups. **G.** The fraction of TME cell infiltration of PDHA1 high and low groups using ssGSEA algorithm. The top end, median line, and bottom end of the box represent the 25%, 50% and 75% value, respectively. the black dots showed outliers. The asterisks illustrated the statistical P value (*P < 0.05, **P < 0.01, ***P < 0.001, ****P < 0.0001, ns P>0.05).


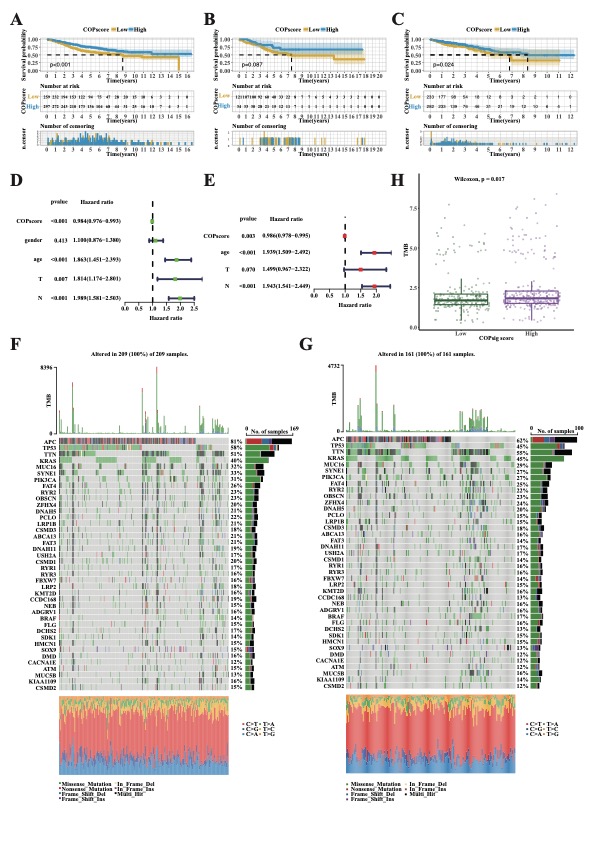


**Figure S3.** Clinical prognosis and mutational significance in high and low COPsig score groups. **A-C.** Survival analysis of COPsig score in collected independent CRC cohort including GSE39582 (**A**), GSE103479 (**B**), and TCGA-CRC (**C**) (Statistical significance were calculated by Log-rank test). **D-E.** Univariate (**D**) and multivariate (**E**) Cox regression analysis for COPsig score in meta-cohort shown by the forest plot. **F-G.** ﻿The waterfall plot of tumor somatic mutation established by those with high COPsig score (**F**) and low COPsig score (**G**). Each column represented an individual patient. The upper barplot showed the tumor mutational burden. The right barplot showed the frequency of each variant type. **H.** Relative distribution of tumor mutational burden in COPsig score high and low groups.

**Figure S4.** the immune cell infiltration characteristics in high and low COPsig score group. **A-B.** ﻿The immune score (**A**) and stromal score (**B**) of two COPsig score groups in meta-cohort. **C-D.** The immune score (**C**) and stromal score (**D**) of two COPsig score groups in ICIs cohort. **E-F.** Heatmap for immune responses based on ssGSEA, MCPcounter, xCell, and EPIC algorithms among high and low COPsig score group in meta-cohort (**E**) and ICIs cohort (**F**).


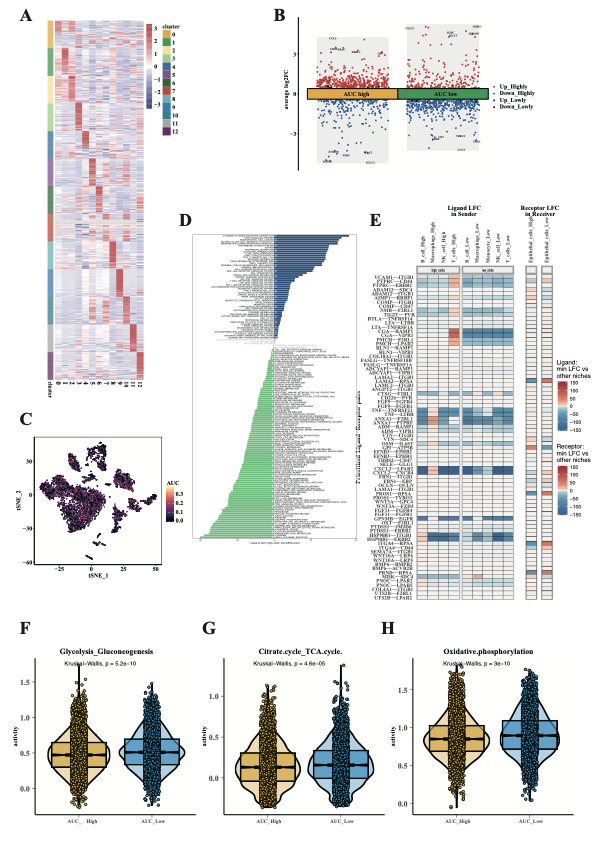


**Figure S5.** Role of cuprotosis signature genes in tumor microenvironment by single cell transcriptome analysis. **A.** heatmap showing expression signatures of top 50 specifically expressed genes in each cluster, the value for each gene is row-scaled Z score. **B.** Differential gene expression analysis showing up- and down-regulated genes across AUCell high and low groups. Highly regulated (an adjusted p value < 0.01) is indicated in red, while an adjusted p value ≥ 0.01 is indicated in black. **C.** t-SNE plots based on the AUCell score of each cell. **D.** Barplot depicting the GSVA score of representative KEGG pathways curated from MSigDB in three cuprotosis patterns (AUCell high vs. AUCell low). **E.** Heatmap showed ligand-receptor interaction in each cell. **F-H.** ﻿Metabolic differences in AUCell high and low groups. **(F)** ﻿Glycolysis_Gluconeogenesis. **(G)** ﻿Citrate.cycle_TCA.cycle. **(H)** ﻿Oxidative.phosphorylation.

**Table S1.** The primer sequences of CRGs

| Primer name | Sequence (5' -> 3') | Length |
| --- | --- | --- |
| FDX1-F | TTCAACCTGTCACCTCATCTTTG | 23 |
| FDX1-R | TGCCAGATCGAGCATGTCATT | 21 |
| LIAS-F | CAGCCCAGTCAGACCGTTAAG | 21 |
| LIAS-R | TTTCTGGCGTTTTAGGTTTCCT | 23 |
| LIPT1-F | CCTCTGTTGTAATTGGTAGGCAT | 23 |
| LIPT1-R | CTGGGGTTGGACAGCATTCAG | 21 |
| DLD-F | CTCATGGCCTACAGGGACTTT | 21 |
| DLD-R | GCATGTTCCACCAAGTGTTTCAT | 23 |
| DLAT-F | CGGAACTCCACGAGTGACC | 19 |
| DLAT-R | CCCCGCCATACCCTGTAGT | 19 |
| PDHA1-F | TGGTAGCATCCCGTAATTTTGC | 22 |
| PDHA1-R | ATTCGGCGTACAGTCTGCATC | 21 |
| PDHB-F | AAGAGGCGCTTTCACTGGAC | 20 |
| PDHB-R | ACTAACCTTGTATGCCCCATCA | 22 |
| MTF1-F | CACAGTCCAGACAACAACATCA | 22 |
| MTF1-R | GCACCAGTCCGTTTTTATCCAC | 22 |
| GLS-F | AGGGTCTGTTACCTAGCTTGG | 21 |
| GLS-R | ACGTTCGCAATCCTGTAGATTT | 22 |
| CDKN2A-F | GATCCAGGTGGGTAGAAGGTC | 21 |
| CDKN2A-R | CCCCTGCAAACTTCGTCCT | 19 |
| GAPDH-F | GGAGCGAGATCCCTCCAAAAT | 21 |
| GAPDH-R | GGCTGTTGTCATACTTCTCATGG | 23 |
